# OsJAZ13 Negatively Regulates Jasmonate Signaling and Activates Hypersensitive Cell Death Response in Rice

**DOI:** 10.3390/ijms21124379

**Published:** 2020-06-19

**Authors:** Xiujing Feng, Lei Zhang, Xiaoli Wei, Yun Zhou, Yan Dai, Zhen Zhu

**Affiliations:** State Key Laboratory of Plant Genomics and National Center for Plant Gene Research (Beijing), Institute of Genetics and Developmental Biology, The Innovative Academy of Seed Design, Chinese Academy of Sciences, Beijing 100101, China; fengxj187@126.com (X.F.); lzhang@genetics.ac.cn (L.Z.); xlwei@genetics.ac.cn (X.W.); zhouyun@genetics.ac.cn (Y.Z.)

**Keywords:** jasmonate, signal transduction, *OsJAZ13*, hypersensitive response, cell death, rice

## Abstract

Jasmonate ZIM-domain (JAZ) proteins belong to the subgroup of TIFY family and act as key regulators of jasmonate (JA) responses in plants. To date, only a few JAZ proteins have been characterized in rice. Here, we report the identification and function of rice *OsJAZ13* gene. The gene encodes three different splice variants: *OsJAZ13a*, *OsJAZ13b*, and *OsJAZ13c*. The expression of *OsJAZ13* was mainly activated in vegetative tissues and transiently responded to JA and ethylene. Subcellular localization analysis indicated OsJAZ13a is a nuclear protein. Yeast two-hybrid assays revealed OsJAZ13a directly interacts with OsMYC2, and also with OsCOI1, in a COR-dependent manner. Furthermore, OsJAZ13a recruited a general co-repressor OsTPL via an adaptor protein OsNINJA. Remarkably, overexpression of *OsJAZ13a* resulted in the attenuation of root by methyl JA. Furthermore, *OsJAZ13a*-overexpressing plants developed lesion mimics in the sheath after approximately 30–45 days of growth. Tillers with necrosis died a few days later. Gene-expression analysis suggested the role of *OsJAZ13* in modulating the expression of JA/ethylene response-related genes to regulate growth and activate hypersensitive cell death. Taken together, these observations describe a novel regulatory mechanism in rice and provide the basis for elucidating the function of *OsJAZ13* in signal transduction and cell death in plants.

## 1. Introduction

Jasmonate (JA) plays a vital role in plant-development regulation, including fertility, root growth, trichome development, anthocyanin accumulation, and senescence, as well as defense mechanisms, including responses to biotic and abiotic stress [1,2,3,4,5]. The JA signaling cascade is broadly conserved in land plants [6,7,8,9]. In the absence of jasmonoyl-l-isoleucine (JA-Ile), JASMONATE ZIM-DOMAIN (JAZ) proteins can interact with the basic helix–loop–helix (bHLH) transcription factor (TF) (e.g., MYC2) or other TFs and repress their activity, thus blocking the expression of JA-responsive genes [10,11]. This repression is mediated by two distinct mechanisms [12]. In one mechanism, MYC-bound JAZs recruit the co-repressor TOPLESS (TPL), either directly [13] or indirectly through the adaptor protein NOVEL INTERACTOR OF JAZ (NINJA) [11]. As another mechanism, JAZ binding to MYC restricts its access to the MED25 co-activator subunit of the mediator complex [14,15,16]. As JA-Ile concentration increases in response to tissue damage or other factors, JAZs interact with the F-box protein CORONATINE INSENSITIVE1 (COI1) and are degraded by the 26S proteasome complex mediated by the SKP1-CUL1-F-box protein (SCF)^COI1^, which releases the TFs interacting with JAZ, and thus activates the transcription of JA-dependent genes [8,17,18,19,20].

Based on the amino acid sequence and evolutionary relationship, JAZs are a subgroup of TIFY superfamily proteins that contain the highly conserved TIFY (TIFF/YXG) or ZIM motif and function as TFs in plants [18,21,22]. Unlike other subgroups of ZIM and PPD proteins, JAZs lack any known TF DNA-binding domains, but possess a conserved TIFY domain and C-terminal Jas domain [21,23]. The former consists of the most highly conserved motif TIF[F/Y]XG, and the latter is characterized by an S-L-X(2)-F-X(2)-K-R-X(2)-R core that is delimited by a conserved N-terminal Pro (P) sequence and a C-terminal Pro-Tyr (PY) sequence [23]. As mentioned above, JAZs function by directly or indirectly interacting with target proteins to regulate downstream signaling. The Jas motif contributes to JAZ binding to the COI1 receptor and the downstream target MYC2 [23]. The conserved TIFY motif mediates the formation of homo-dimers and hetero-dimers between JAZ proteins, and is also responsible for the binding of NINJA protein, which harbors an ERF-associated amphiphilic repression (EAR) motif and mediates the interaction with TPL [11,24,25]. Notably, the EAR motif (LXLXLX or DLNXXP, in which X can be any amino acid) functions in gene repression [26] and is also found in some JAZ members. For example, in *Arabidopsis*, JAZ8 and JAZ13 directly interact with TPL, as has been demonstrated by using the yeast two-hybrid (Y2H) assay [13,27]. In addition, JAZ function with HDA6 and EIN3 to regulate ERF and ABRE target genes via epigenetic modification [28,29].

Growing evidence suggests that *JAZ* genes play important roles in plant development and defense responses. The majority of functional studies of JAZ proteins have mainly focused on *Arabidopsis* [30,31,32,33,34] and tobacco [35,36,37]. The rice genome encodes 15 *JAZ* family members [38,39]; however, only a few of the encoding genes have been functionally characterized to date [40,41,42,43,44,45]. For example, OsJAZ1 interacts with OsbHLH148 to enhance drought resistance, and it also interacts with OsMYC2 to regulate spikelet development [42]. Furthermore, overexpression of *OsJAZ8* enhances resistance to bacterial blight in rice [45], whereas *OsJAZ9* modulates salt stress tolerance [43]. In addition, rice JAZ protein (OsJAZ9 or OsJAZ11) can bind to complex of RSS3-bHLH to regulate JA-responsive genes and root cell elongation [46].

A previous report demonstrated that *OsJAZ13* (*OsTIFY11e*) was more strongly induced by JA, and also strongly induced by at least two stress treatments ( salt and cold) [38]; based on these results, in the current study, we focused on OsJAZ13 and characterized its function in rice. The gene encodes three splice variants. OsJAZ13 functions as a nuclear protein. We report that OsJAZ13 physically interacted with OsMYC2 to block its activity, perhaps by recruiting the general co-repressor OsTPL via the adaptor protein OsNINJA. In addition, OsJAZ13a interacted with OsCOI1 and was degraded by the SCF^COI1^ complex-mediated 26S proteasome in a coronatine (COR, a mimic of JA)-dependent manner. Furthermore, transgenic rice plants overexpressing *OsJAZ13a* exhibited a JA-insensitive phenotype when treated with methyl jasmonate (MeJA) and developed spontaneous necrotic lesions on the sheath at the tillering stage. The expression of some JA/ethylene (ET) pathway-related genes, hypersensitive response marker genes, and defense-related genes was altered in the *OsJAZ13*-overexpressing rice plants. Based on these observations, we conclude that OsJAZ13 acts as a repressor of JA signaling by forming a transcriptional regulation complex with OsMYC2, which further recruits the co-repressor OsTPL through the interaction of OsJAZ13 and OsNINJA to regulate the expression of JA/ethylene response-related genes and activate hypersensitive cell death in rice.

## 2. Results

### 2.1. Isolation and Identification of OsJAZ13

**Figure 1:**
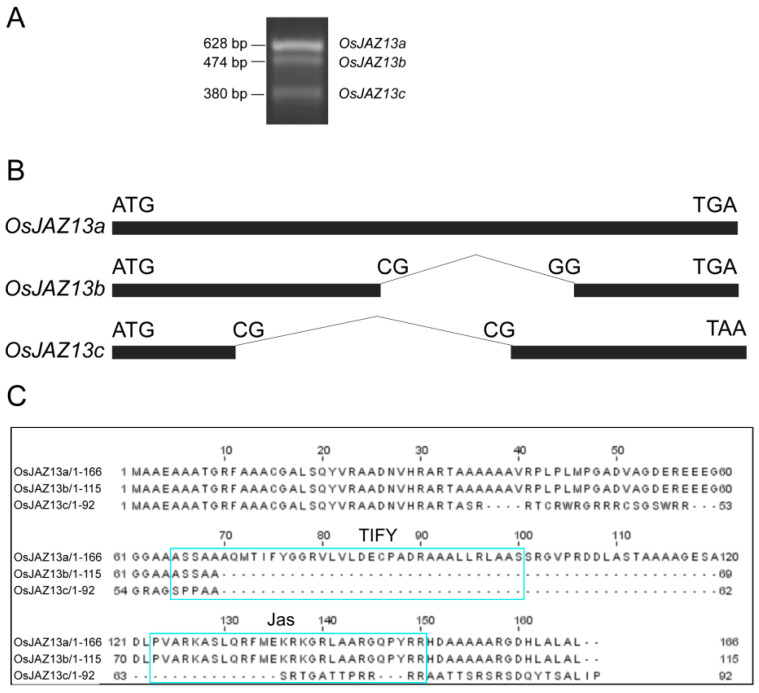
Identification of three alternative splicing variants of *OsJAZ13*. (**A**) RT-PCR analysis of *OsJAZ13* transcripts using RNAs isolated from MH86 at the grain filling stage. This experiment was performed for at least three independent biological repeats. (**B**) Schematic diagram of alternatively spliced transcripts *OsJAZ13a*, *OsJAZ13b*, and *OsJAZ13c*, respectively. Blank box represented exons. (**C**) Alternative splicing differentially affects domain distribution in the three OsJAZ13 splice variants.

Rice genome reportedly encodes 15 proteins from the JAZ family, which have different cellular functions [38]. In the current study, we used RT-PCR to isolate cDNA of the *OsJAZ13* gene from rice leaves. We obtained three different amplified cDNA fragments, using gene-specific primer pairs (Supplementary Table S1) designed based on the sequence of the *OsJAZ13* gene (*LOC_Os10g25230*) available from the TIGR database (http://www.tigr.org) (Figure 1A). Cloning and sequencing revealed that the three cDNAs were 628, 474, and 380 bp long. We designated them as *OsJAZ13a*, *OsJAZ13b*, and *OaJAZ13c*, respectively (Figure 1A). Comparison of the three splice variants of *OsJAZ13* with the genomic sequence revealed that *OsJAZ13a* lacked an intron, and that unusual splice sites, CG-GG and CG-CG, were present in *OsJAZ13b* and *OsJAZ13c*, respectively (Figure 1B). The *OsJAZ13a* cDNA sequence contained an open reading frame (ORF) of 501 bp encoding a 166-residue protein that harbors the conserved TIFY domain in the middle section and a C-terminal Jas domain (Figure 1B,C). Compared with the *OsJAZ13a* ORF, *OsJAZ13b* lacked nucleotides 209–361, which resulted in a nearly complete deletion of the entire TIFY domain (Figure 1B,C). Furthermore, *OsJAZ13c* ORF lacked a 270-bp fragment in the middle region, which resulted in a complete loss of the TIFY and Jas domains. This splicing event also caused a frame-shift mutation, which added seven amino acids to the protein C-terminus (Figure 1B,C).

A phylogenetic tree was generated with the putative amino acid sequences of 15 rice JAZ proteins, three *Arabidopsis* JAZ proteins, one tomato JAZprotein, and one tobacco JAZ protein. The 20 JAZ proteins were aligned by using Clustal W 1.8. The analysis revealed the presence of the conserved TIFY and Jas domains (Figure S1A). Furthermore, OsJAZ13 and OsJAZ15 clustered in one branch, and OsJAZ13 shared 40% amino acid identity with AtJAZ6 (Figure S1B).

### 2.2. OsJAZ13 Expression Pattern

**Figure 2:**
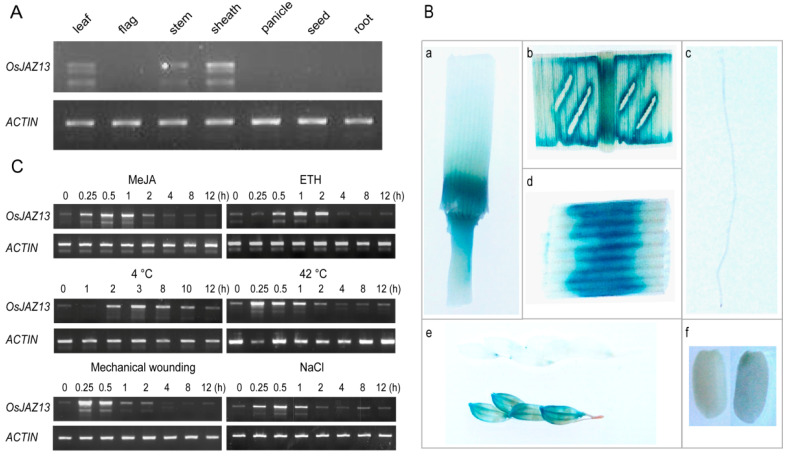
Expression pattern analysis of *OsJAZ13*. (**A**) The relative expression of *OsJAZ13* in various tissues. RNA was extracted from different organs of MH86, at the seedling and filling stage. (**B**) Expression patterns of *OsJAZ13* indicated by β-glucuronidase (GUS) staining. Samples at seedling, flowering, and filling stages were collected, stained, and then photographed with a SONY camera. (**a**) Stem, (**b**) leaf, (**c**) root, (**d**) sheath, (**e**) panicle, and (**f**) seed. (**C**) Three-leaf-stage seedlings of MH86 were treated with 100 µM MeJA (0.1% DMSO dissolved and as mock treatment), 100 µM ethephon (ddH_2_O dissolved and as mock treatment), 4 °C, 42 °C, mechanical wounding, and 200 mM NaCl. Various numbers in this figure represent different time points for sample treatment. Samples were collected at indicated times. *ACTIN* served as internal control. All experiments were performed for at least three independent biological repeats.

To investigate the expression pattern of the *OsJAZ13* gene, we performed semi-quantitative RT-PCR, using total RNA isolated from various tissues and organs of MH86 rice. As shown in Figure 2A, *OsJAZ13* was mainly expressed in the leaf, sheath, and stem, but not in the seed and root. The expression pattern was confirmed by the analysis of transgenic plants harboring the construct of the native *OsJAZ13* promoter fused with the *GUS* gene. In line with gene-expression analysis, GUS activity was detected in the vegetative organs leaf and stem, especially in the sheath, but not in the seed, root, and anther (Figure 2B).

Furthermore, we investigated the responses of *OsJAZ13* expression to phytohormones and abiotic stress. Rice seedlings at the three-leaf stage were treated with exogenous hormones MeJA or ETH and were exposed to various abiotic stresses, such as cold temperature (4 °C), heat (42 °C), NaCl, and mechanical wounding. *OsJAZ13* expression was rapidly induced under the above conditions (Figure 2C). Notably, when the plants were treated with MeJA and ETH, *OsJAZ13* transcripts accumulated transiently, reaching a peak level at 0.5 and 2 h, and subsiding to near-basal levels at 4 h (Figure 2C). Taken together, these results indicate that *OsJAZ13* is responsive to JA/ET signaling and certain abiotic stresses.

### 2.3. Subcellular Localization of OsJAZ13

**Figure 3:**
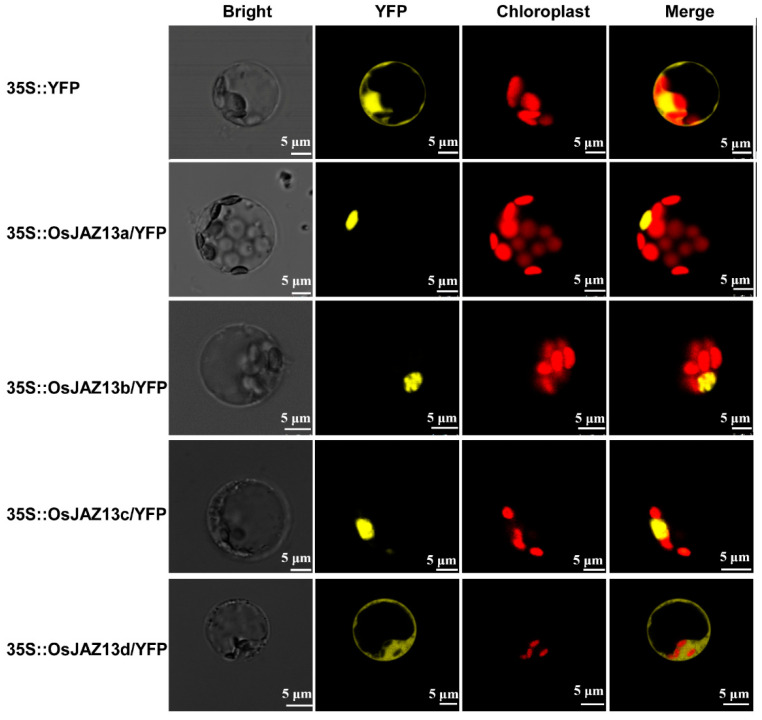
Subcellular localization of OsJAZ13 splice variants. OsJAZ13s-YFP fusion proteins (*pAVA321/35S:: YFP*, *35S:: OsJAZ13a/YFP*, *35S:: OsJAZ13b/YFP*, *35S:: OsJAZ13c/YFP*, and *35S:: OsJAZ13d* (amino acids 1 to 110 of OsJAZ13)*/YFP*) were transiently expressed in rice protoplast. YFP fluorescence was detected, using a confocal laser, after incubation for 24–48 h, in the dark, at 28 °C. Scale bars = 5 μm. This experiment was performed for three independent biological repeats.

To determine the subcellular localization of OsJAZ13, we transiently expressed fusion proteins of YFP and the three different OsJAZ13 variants under the control of the 35S promoter in rice protoplast. Subcellular localization analysis revealed that the three fusion proteins were specifically targeted to the nucleus, whereas the control YFP protein was present both in the nucleus and cytoplasm (Figure 3). It was previously shown that JAZ protein lacking the Jas domain mainly localizes in the nucleus [25]. Therefore, we also investigated the subcellular localization of a truncated isoform of OsJAZ13 (OsJAZ13d, which lacks the Jas motif). Unexpectedly, the fluorescent signal was localized both in the nucleus and cytoplasm (Figure 3), which was inconsistent with the previous report [25,47].

### 2.4. OsJAZ13 Interacts with the Key Regulators of JA Signaling Pathway

**Figure 4:**
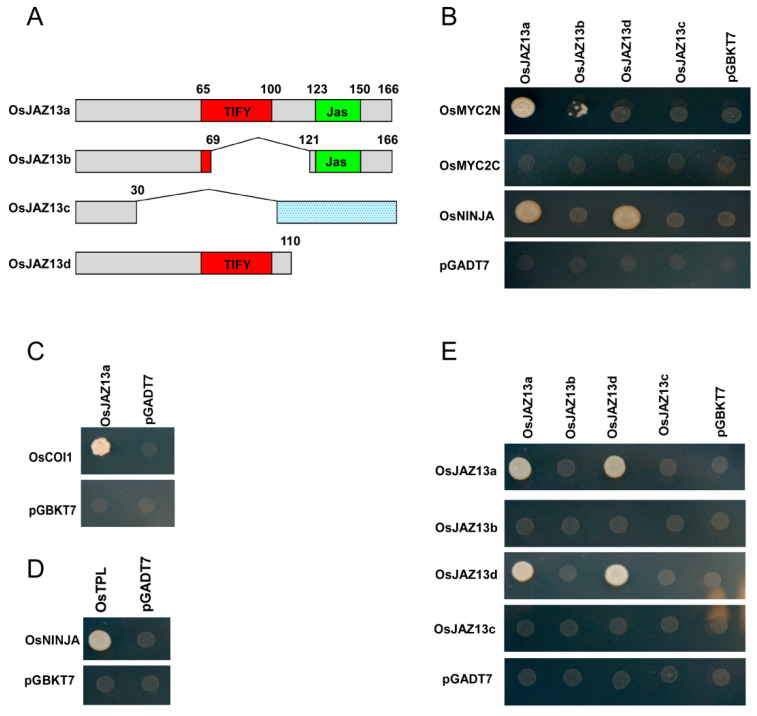
Y2H assay of the interactions between OsJAZ13 and the key regulators of the JA signaling pathway. (**A**) Schematic diagram of OsJAZ13 splice variants and the truncated derivative. The diagram displays the highly conserved Jas (green) and TIFY (red) domains, as well as the weakly conserved sequence (gray). The indicated combinations of plasmids were cotransformed into the yeast reporter strain Y2H gold; successfully transformed colonies were identified on appropriate selective minimal agar. (**B**) Interaction of OsJAZ13a, OsJAZ13b, OsJAZ13c, and OsJAZ13d with OsMYC2 derivatives or OsNINJA in the Y2H system. (**C**) Coronatine-dependent interaction of OsJAZ13a with OsCOI1 in the Y2H system. (**D**) Interaction between OsNINJA and OsTPL in the Y2H system. (**E**) Y2H analysis of the dimerization of OsJAZ13 splice variants (OsJAZ13a, OsJAZ13b, OsJAZ13c) and the truncated derivative OsJAZ13d. The empty bait (pGBKT7) and prey (pGADT7) vectors were used as negative controls. This experiment was performed for at least three independent biological repeats.

We next examined the interaction of the three different OsJAZ13 isoforms and the truncated isoform OsJAZ13d with rice homologs of OsMYC2 and OsCOI1, using the Y2H system (Figure 4A). Because pGBDK-T7-OsMYC2 showed self-activation (data not shown), we split OsMYC2 into two sections, the N-terminal domain (OsMYC2N; amino acids 1 to 342) and the C-terminal domain (OsMYC2C; amino acids 282 to 700), accordingly. Analysis of protein–protein interactions revealed that OsJAZ13a isoform interacted strongly with OsMYC2N, while OsJAZ13b interacted very weakly with OsMYC2N, but not with OsMYC2C. This indicated that the Jas domain of OsJAZ13 and the N-terminal portion of OsMYC2 are necessary for the interaction between OsJAZ13 and OsMYC2 (Figure 4B). Furthermore, OsJAZ13a was bound by OsCOI1 in the presence of 30 μM COR (Figure 4C).

We further evaluated the interaction between OsJAZ13, OsNINJA, and OsTPL. The analysis indicated that OsNINJA interacted with OsJAZ13a and OsJAZ13d via the TIFY motif, and the interaction between OsNINJA and OsTPL indirectly tethered OsJAZ13 to OsTPL (Figure 4D). In addition, we investigated the interaction of three alternative splicing isoforms (OsJAZ13a, OsJAZ13b, and OsJAZ13c) and OsJAZ13d, with each other and themselves. The analysis revealed that both OsJAZ13a and OsJAZ13d formed homo-dimers, whereas neither OsJAZ13b nor OsJAZ13c were involved in any interaction (Figure 4E). This suggests that the TIFY domain is responsible for homo-dimer formation.

### 2.5. OsJAZ13 Negatively Regulates JA Response

**Figure 5:**
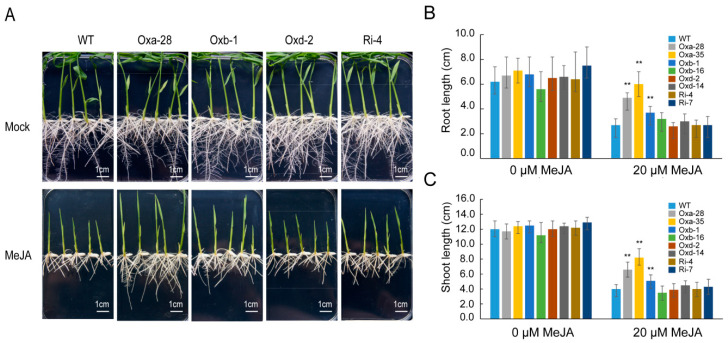
*OsJAZ13* functions in jasmonate signaling. (**A**) Overexpression of *OsJAZ13a* exhibited the increased JA-insensitivity phenotype, and *OsJAZ13b* showed in slight JA-insensitivity; *OsJAZ13d* and suppression of *OsJAZ13* resulted in JA-sensitivity phenotype similar to wild-type plants. The photograph indicated the phenotypes of seven-day-old seedlings of wild-type (WT) and transgenic lines grown on MS medium with 20 μM MeJA or under normal conditions. Scale bars = 1 cm. (**B,C**) Quantification of MeJA-induced growth inhibition of transgenic and WT plants. Data are the mean ± SD (*n* = 10 seedlings per genotype). Asterisks indicate significant differences in root and shoot length (^**^ *p* < 0.01, Student’s *t*-test) in comparisons with WT grown in the absence or presence of MeJA. Oxa-28 and Oxa-35 represent overexpression lines of *OsJAZ13a*; Oxb-1 and Oxb-16 represent overexpression lines of *OsJAZ13b*; Oxd-2 and Oxd-14 represent overexpression lines of *OsJAZ13d*; and Ri-4 and Ri-7 represent antisense suppression lines of *OsJAZ13*. All experiments were performed for three independent biological repeats.

To investigate the effect of *OsJAZ13* overexpression or antisense suppression on the JA signal transduction pathway, representative T_1_ heterozygous overexpression line for *OsJAZ13a*, homozygous overexpression lines for *OsJAZ13b* and *OsJAZ13d*, and a homozygous antisense suppression line were analyzed in root growth inhibition assays. Unexpectedly, *Ubi-OsJAZ13a* seedlings (Oxa-28) exhibited the least-sensitive phenotype to JA, *Ubi-OsJAZ13b* (Oxb-1) showed slight insensitivity, and *Ubi-OsJAZ13d* (Oxd-2) and *OsJAZ13* antisense suppression (Ri-4) transgenic seedlings showed sensitivity similar to that of the wild type to exogenous MeJA treatment (Figure 5A). The root and shoot lengths of *Ubi-OsJAZ13*a (*n* = 10) were significantly different from those of the wild type and other transgenic plants (Figure 5B,C).

**Figure 6:**
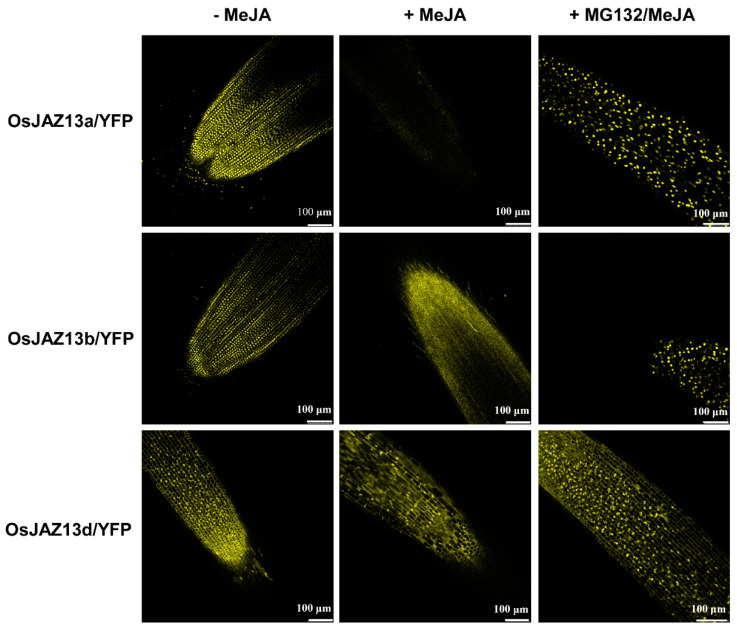
Stability of OsJAZ13 splice variants in vivo. Transgenic rice seedlings expressing *Ubi-OsJAZ13a-YFP*, *Ubi-OsJAZ13b-YFP*, and *Ubi-OsJAZ13d-YFP* grown in 1/2MS medium treated with water (−MeJA) or 50 μM MeJA (+MeJA) for 2 h, or 100 μM MG132 + 50 μM MeJA (+MG132/MeJA) for 2 h. A confocal laser scanning microscopy was used to observed the YFP signal in root tissue. This experiment was performed for three independent biological repeats. Scale bars = 100 µm.

To determine whether the different root growth response of the distinct genotypes was associated with protein stability in vivo, we compared JA-mediated changes in the fluorescence of different alternative splicing isoforms of *OsJAZ13s-YFP* that were stably expressed in rice, using confocal laser scanning microscopy. In the absence of JA treatment, nucleus-localized YFP signals were observed for *Ubi-OsJAZ13a-YFP* and *Ubi-OsJAZ13b-YFP* (Figure 6). After MeJA treatment, the nucleus-localized YFP signals in the roots of *Ubi-OsJAZ13a-YFP* and *Ubi-OsJAZ13b-YFP* seedlings largely disappeared, and this effect was partially inhibited by pretreating the seedlings with the MG132, a 26S proteasome inhibitor. By contrast, the YFP signals were detected in the roots of *Ubi-OsJAZ13d-YFP* seedlings before and after treatment. These observations indicate that OsJAZ13a and OsJAZ13b are more sensitive to JA-mediated degradation in vivo than OsJAZ13d.

### 2.6. OsJAZ13a Overexpression Affects the Expression of JA-Signaling-Related Genes

**Figure 7:**
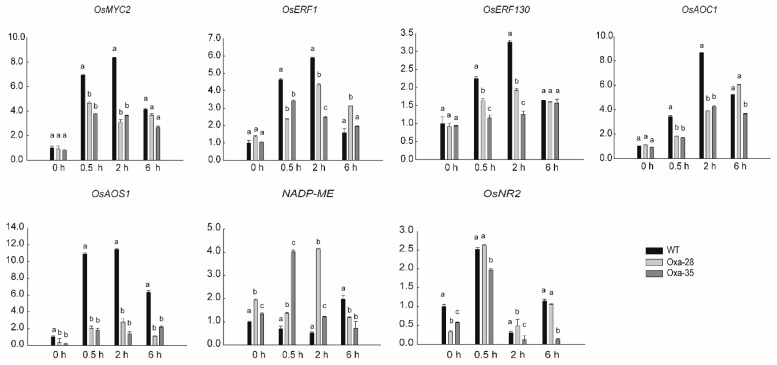
Expression analysis of JA-signaling-related genes in *OsJAZ13a*-overexpression plants treated with MeJA by qRT-PCR. RNA samples were collected at the indicated time from three-leaf-stage seedlings of wild-type and *OsJAZ13a*-overexpression lines treated with 100 µM MeJA. *ACTIN* served as an internal control. The values are means ± SD (*n* = 3 replicates). Different lowercase letters indicate significant differences (*p* < 0.05). The *y*-axis represents the relative expression level. This experiment was performed for three independent biological repeats.

JA and ET signals can interact synergistically or antagonistically to affect various aspects of plant growth development and defense [48,49]. To identify candidate genes that might be regulated by OsJAZ13, and that are required for plant growth and defense, genes related to the JA/ET synthesis/signaling, such as ones encoding allene oxide synthase (*OsAOS1*, *LOC_Os03g5580*0), allene oxide cyclase (O*sAOC1*, *LOC_Os03g32314*), *OsMYC2*, *OsERF1* (*LOC_Os04g46220*), and *OsERF130* (*LOC_Os05g41760*), or JA/ET-defense-regulated genes, such as ones encoding NADH/NADPH dependent NO_3_^−^ reductase (*OsNR2*, *LOC_Os02g53130*) and NADP-malic enzyme (*NADP-ME*, *LOC_Os01g54030*), were tested in *OsJAZ13a*-overexpressing and wild-type plants sprayed with 100 μM MeJA. RT-qPCR analysis indicated that, compared with the wild type, the expression of *OsMYC2*, *OsERF1*, *OsERF130*, *OsAOC1*, and *OsAOS1* was dramatically impaired in the *OsJAZ13a*-overexpressing lines 0.5 and 2 h after treatment (Figure 7). Furthermore, *NADP-ME* expression was obviously induced relative to the wild type, and *OsNR2* expression was obviously suppressed in untreated transgenic plants. These findings corroborate the notion that OsJAZ13 negatively regulates JA response.

### 2.7. OsJAZ13a Overexpression Leads to Hypersensitive Cell Death Under Lesion-Inducing Conditions

**Figure 8:**
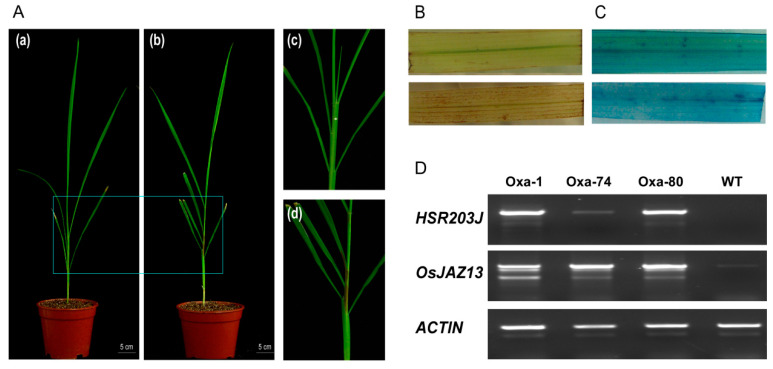
*OsJAZ13a*-overexpression plants develop spontaneous necrosis on the sheath, which accumulates ROS and high expression of hypersensitive response marker gene. (**A**) Necrotic spots on sheath of *OsJAZ13a*-overexpression plants indicated by WT (**a**) and Oxa (**b**). Scale bars = 5 cm. Magnified views of the WT (**c**) and Oxa (**d**) are shown in the right panels. The plants were grown in a greenhouse (25 °C, 55–65% humidity, 14 h light/10 h dark) for 30–45 days. (**B**) Detection of H_2_O_2_ content in the leaves of *OsJAZ13a*-overexpression lines while lesion appearing on the sheath, WT (above) and Oxa (bottom). The brown region staining by DAB on leaves indicates the H_2_O_2_ level. (**C**) Detection of PCD in the leaves of *OsJAZ13a*-overexpression lines. The blue spots on leaves by typan blue staining indicate cell death, WT (above) and Oxa (bottom). (**D**) Expression analysis of *OsJAZ13* and hypersensitive response marker gene *HSR203J* by RT-PCR; RNA was extracted from leaves of *OsJAZ13a*-overexpression plants when lesion emerged in the sheath. *ACTIN* served as internal control. These experiments were performed for three independent biological repeats.

When *OsJAZ13a*-overexpressing plants were grown in the greenhouse, at a humidity of 55–65% and illumination of 14 h light/10 h dark, lesion mimics gradually emerged in the sheath after growth for approximately 30–45 days, even if the soil was sterilized to prevent infection with pathogenic bacteria (Figure 8A). Notably, no pathogenic bacteria or fungi were isolated from the lesion mimics. Such necrotic transgenic plants died a few days later, with none surviving to the next development phase, whereas the wild-type plants grew well. To verify the assumption of hypersensitive reaction, we examined whether lesion mimics in the transgenic plant sheath triggered the production of ROS and PCD. DAB staining revealed a higher accumulation of H_2_O_2_ in the leaves of *OsJAZ13a*-overexpressing transgenic lines than in the wild type (Figure 8B), suggesting occurrence of an oxidative burst in the former. Furthermore, trypan blue staining indicated concurrent PCD (Figure 8C). RT-PCR analyses showed that *HSR203J* (*LOC_Os05g33940*), a marker gene in hypersensitive response, was drastically upregulated in overexpressing lines, compared with the wild type (Figure 8D). We therefore speculate that *OsJAZ13* might be necessary for the induction or propagation of ROS and for the occurrence of PCD.

### 2.8. Gene Expression during Necrosis Development in OsJAZ13a-Overexpressing Plants

**Figure 9:**
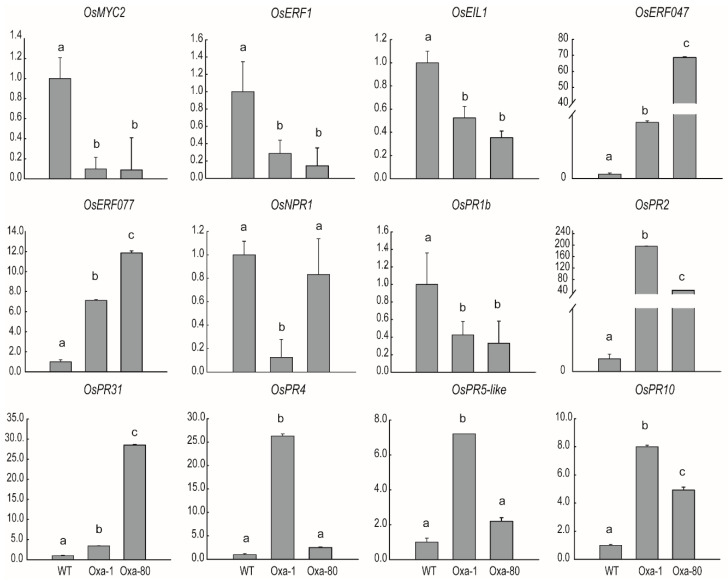
Expression analysis of necrosis-development-related genes in *OsJAZ13a*-overexpression lines. RNA was extracted from rice leaves of *OsJAZ13a* overexpression lines and wild type, and which were planted in greenhouse and lesion appeared on the sheath of transgenic plants. We performed qRT-PCR to analyze associated genes involved in JA/ET signaling pathway *OsMYC2*, *OsERF1*, *OsEIL1*, *OsERF047*, and *OsERF077*; pathogen-defense genes *OsNPR1* and *OsPR1b*; and defense-related genes *OsPR2*, *OsPR31*, *OsPR4, OsPR5-like*, and *OsPR10*. *ACTIN* served as the internal control. The values are means ± SD (*n* = 3 replicates). Different lowercase letters indicate significant differences (*p* < 0.05). The *y*-axis represents the relative expression level. This experiment was performed for three independent biological repeats.

To elucidate the possible mechanisms of sheath necrosis in the *OsJAZ13a*-overexpressing plants, we determined the expression of a subset of related genes that could have been involved in this phenotype (Figure 9). Accordingly, RT-qPCR analysis revealed that the expression of *OsMYC2* was obviously suppressed in the *OsJAZ13a*-overexpressing lines, with transcript levels nine- and six-fold lower than those in the wild-type plants. In addition, the expression of *OsERF1* (*LOC_Os04g46220*), *OsEIL1* (*LOC_Os03g020790*), *OsNPR1 (LOC_Os01g09800*), and *OsPR1b* (*LOC_Os01g28450*) was also reduced in the transgenic plants. Furthermore, the expression of ET-signaling-pathway genes *OsERF047* (*LOC_Os03g09170*) and *OsERF077* (*LOC_Os04g52090*) was significantly upregulated in the transgenic lines, 56- and 11-fold, respectively. Furthermore, the expression of defense-related genes *OsPR2* (*LOC_Os01g71820*), *OsPR31* (*LOC_Os01g47070*), *OsPR4* (*LOC_Os11g37950*), *OsPR5-like* (*LOC_Os03g46060*), and *OsPR10* (*LOC_Os12g36880*) was upregulated (Figure 9). These observations imply that the phenotype of transgenic rice was related to the ET/JA signaling pathway.

### 2.9. Paddy Field Performance of OsJAZ13a-Overexpressing Plants

**Figure 10:**
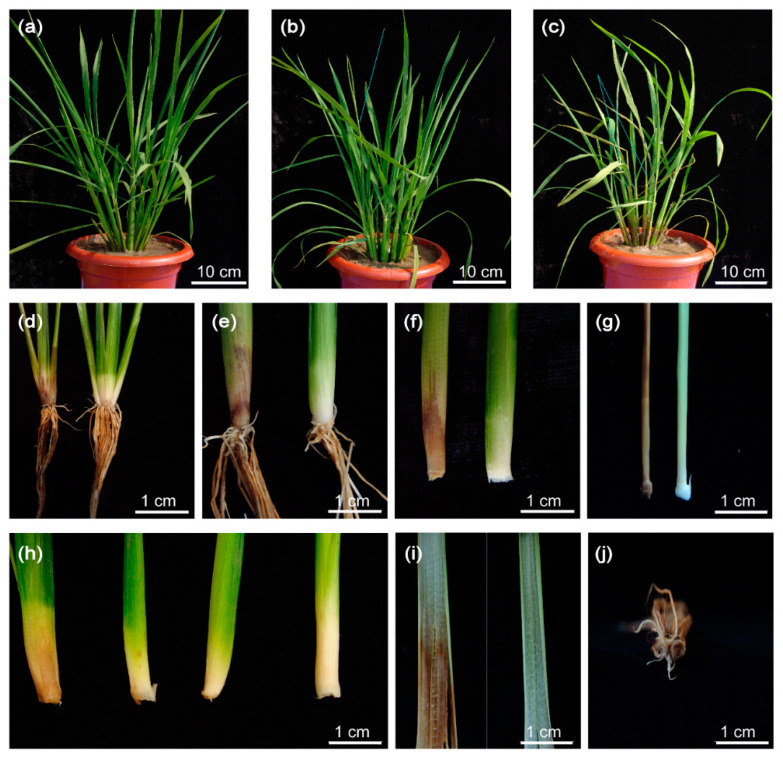
Phenotype analysis of *OsJAZ13a*-overexpression plants under paddy fields. *OsJAZ13a*-overexpression lines and the wild type were transplanted to paddy fields about 30–45 days till plants developed to the tillering stage. (**a**) Wild type, (**b**) one tiller died, (**c**) two tillers died, (**d**–**g**) comparation of the sheath between dead and normal tillers, (**h**) differential level of the water-soaked tiller, (**i**) the comparison of leaves, and (**j**) the cross-section of a dead tiller. This experiment was performed for three independent biological repeats. Scale bars = 10 cm (a–c) and 1 cm (d–j).

Finally, we investigated the phenotype of *OsJAZ13a*-overexpressing transgenic plants in a paddy field. Compared with *OsJAZ13a*-overexpressing transgenic plants grown in the greenhouse, most of the transgenic plants exhibited water-soaked spots at the bottom of one or two tillers, approximately 30–45 days after transplantation to a paddy field (Figure 10). The spots spread gradually, which led to gradual tiller wilting and death. The remaining tillers grew normally until seed setting. Furthermore, the phenotype was dose-dependent, i.e., more pronounced with a higher *OsJAZ13* expression, suggesting that *OsJAZ13a*-overexpressing lines are susceptible to the ever-changing outdoor conditions that may be associated with humidity.

## 3. Discussion

In the current study, we aimed to investigate the function of OsJAZ13 in rice. We showed that OsJAZ13 has three different alternative splice variants (Figure 1) and functions as a negative regulator of JA signaling in rice (Figure 4, Figure 5 and Figure 6). The expression of *OsJAZ13* was induced by different phytohormones and abiotic stress (Figure 2), indicating it might play an important role in the defense system of rice. We also found that OsJAZ13a-overexpressing plants developed lesion mimics in the rice sheath and activated hypersensitive cell death (Figure 8 and Figure 10), accompanied by altered expression of the JA/ET-related genes and programmed-cell-death genes (Figure 9). These observations demonstrate that OsJAZ13 functions as a repressor in signal transduction and plays an important role in cell death in plants.

### 3.1. Alternative Splicing of OsJAZ13

The ever-changing environmental conditions pose a major challenge to plant survival. Plants preferentially rely on alternative splicing (AS) to cope with such conditions. According to genome-wide analysis, 42–61% of genes containing intron in plant are alternatively spliced [50,51]. Furthermore, published studies also indicate that biotic and abiotic stresses promote the generation of new transcripts to enhance plant fitness [50,52,53,54,55,56]. In the current study, we identified the rice *OsJAZ13* gene, which has three alternative splicing variants (Figure 1). Sequence analysis revealed the presence of unusual splice sites (CG-GG/CG-CG) in the splicing isoforms, which deviated from GT-AG, the classical splicing ruler [57]. This suggested that *OsJAZ13* functions in a novel way to transduce complicated cues and fine-tune the balance between growth and defense. Consistent with these findings, some JAZ family members from diverse plant species also underwent AS phenomena [58]. In *Arabidopsis*, AS leads to a complete or partial deletion of the Jas domain in *JAZ1*, *JAZ3*, *JAZ4*, *JAZ6*, *JAZ9*, *JAZ10*, and *JAZ12* [58]. The alternative splicing of JAZ mediated desensitization of JA signaling through cryptic MYC-interaction N-terminal domain [59]. Overexpression of splice variants of *JAZ2.2*, *JAZ10.3*, and *JAZ10.4* modulates the JA signal output in a dominant-negative fashion, and results in JA insensitivity [23,25,58]. Similarly, in tobacco, *NaJAZc.2* and *NaJAZk.2* undergo editing and loss of the Jas domain [35]. Taken together, AS of *OsJAZ13* broadens the protein repertoire in rice and enhances plant flexibility to resist environmental stress.

### 3.2. Negative Regulation of JA Signal Transduction Pathway by OsJAZ13 Proteins

To explore the molecular mechanism of *OsJAZ13* activity, we investigated the interaction of different isoforms of OsJAZ13 with themselves or other components of the JA signaling pathway. We found that OsJAZ13a and OsJAZ13b interacted with OsMYC2, and that the Jas domain of OsJAZ13 and the N-terminal portion of OsMYC2 were required for the interaction (Figure 4B). Furthermore, OsJAZ13a interacted with OsCOI1 in the presence of COR (Figure 4C). OsJAZ13a and OsJAZ13d formed homo-dimers, and the conserved TIFY domain was involved in these interactions (Figure 4E). These observations are consistent with previous reports in *Arabidopsis* that JAZ proteins form homo- and hetero-dimers, that TIFY domain is essential for these interactions [24,58], and that JAZ proteins function by interacting with MYC2 to repress JA-responsive genes [6].

JAZ proteins recruit the co-repressor TPL via the adaptor protein NINJA, to repress the JA signal transduction pathway [11]. We showed here the interaction between OsJAZ13 and OsNINJA, and that OsNINJA interacted with OsTPL (Figure 4B,E). Collectively, on the one hand, these observations suggest that OsJAZ13 may function as a homo-dimer to interact with OsMYC2 and OsCOI1, and to repress JA signaling. On the other hand, OsJAZ13 interacts with OsNINJA and then recruits OsTPL to exert a suppressive role.

The analysis of protein degradation in vivo revealed that MeJA treatment resulted in YFP signal disappearance from transgenic plants overexpressing *OsJAZ13a* and *OsJAZ13b* (lacking the TIFY motif), but had no effect on transgenic plants overexpressing *OsJAZ13d* (lacking the Jas motif) (Figure 6). This suggested that the degradation of OsJAZ13 was associated with the Jas motif, not the TIFY motif, which is consistent with a previous report [23].

We also showed that overexpression of full-length *OsJAZ13a* cDNA led to the insensitivity of root growth to MeJA treatment (Figure 5A). Such JA insensitive phenotype was also reported for *Arabidopsis* overexpressing full-length cDNA of *GsTIFY10*, *AtJAZ8*, and *ZmJAZ14* [13,60]. According to previous reports, some JA signaling pathway mutants, such as *myc2*, *jar1*, and *coi1*, display a similar insensitive response to root growth inhibition [17,61,62]. Furthermore, in the current study, RT-qPCR analysis revealed that the master genes involved in JA biosynthesis and signal transduction (*OsAOS1*, *OsAOC1*, and *OsMYC2*) were dramatically suppressed in *OsJAZ13a*-overexpressing plants upon MeJA treatment (Figure 7). These findings indicate that the JA-insensitivity phenotype of *OsJAZ13a*-overexpressing plants is associated with blocked JA biosynthesis and signal transduction pathway, as a result of suppressed expression of the key genes involved.

### 3.3. OsJAZ13 Integrates JA and ET Signals to Regulate Hypersensitive Cell Death in Rice

*OsJAZ13a*-overexpressing plants developed spontaneous lesions. This resembled a hypersensitive response induced by pathogens (Figure 8 and Figure 10), although we failed to isolate any pathogenic bacteria or fungi from these lesions. Such lesion mimic phenotype has been observed in many mutants in multi-signaling pathways, including the JA signaling pathway [63,64,65]. For example, JA signaling mutants *fad3/7/8* and *jar1* develop lesions when exposed to O^3^, which blocks JA biosynthesis and signal transduction, as well as O^3^-induced cell death [66]. Most JA-insensitive mutants do not limit the spread of cell death, thus leading to the serious damage of leaf tissue in response to oxidative stress [67].

ROS accumulates in plant cells during plant PCD [68,69,70]. It is a defense mechanism against pathogens or herbivores [71,72,73]. In the current study, accumulation of H_2_O_2_ was indeed detected in *OsJAZ13a*-overexpressing leaves upon lesion emergence in the sheath in greenhouse-grown plants (Figure 8B). In addition, ROS are produced by NADPH oxidases, which can be provided by NADP-ME reaction after MeJA induction [74,75]. We also showed that the expression of *NADP-ME* gene was upregulated upon MeJA treatment (Figure 7), suggesting that it might contribute to the accumulation of H_2_O_2_ in *OsJAZ13a*-overexpressing leaf.

According to previous reports, JAZ proteins function as transcriptional repressors by interacting with the TF MYC2, which regulates gene expression by binding to the G-box in the target gene promoter region, resulting in a negative regulation of defense response genes and a positive regulation of wound response genes [28,61,76]. In *Arabidopsis*, susceptibility to necrogenic pathogen attack and triggered cell death are elevated in *myc2* mutant (*jin1*) [77]. The Y2H analysis presented herein revealed that *OsJAZ13a* interacts with *OsMYC2* (Figure 4B), and RT-qPCR analysis revealed that *OsMYC2* expression was dramatically decreased in a leaf with sheath lesions in *OsJAZ13a*-overexpressing transgenic plants (Figure 9). These observations indicate that the occurrence of lesions in *OsJAZ13a*-overexpressing plants might be associated with a decreased expression of *OsMYC2*.

*ERF1*, encoding one member of the B-3/IX subgroup of ERF family, is an early ET- and JA-responsive gene and functions to integrate both signals to activate ET/JA-dependent responses to pathogens [78]. Constitutive expression of *ERF1* confers resistance to necrotrophic fungi in *Arabidopsis* [79]. Furthermore, *ERF1* is positively regulated by EIN3/EIL1. In *Arabidopsis*, JAZ1 interacts with EIN3/EIL1 and also represses transcription of the encoding gene [28]. In the current study, overexpression of *OsJAZ13a* drastically suppressed the expression of *OsERF1* (3–7-fold change) and *OsEIL1* (2–3-fold change) during lesion development. Therefore, the lesion phenotype in *OsJAZ13a*-overexpressing plants can be attributed to the decreased expression of *OsERF1* and *OsEIL1*. In addition, we analyzed the expression of another *ERF* gene, *OsERF077*, which encodes a protein from the VIII-b subgroup family and harbors the EAR motif, and whose transient overexpression in tobacco leaf results in cell death [80]. Consistent with the previous report, we showed that *OsERF077* expression was dramatically upregulated in *OsJAZ13a*-overexpressing plants upon lesion emergence (Figure 9).

Apart from impacting the expression of early response genes to resist an exogenous attack, the expression of pathogenesis-related (PR) protein encoding genes was subsequently activated, by MYC2 or ERF binding to the promoter region. In *Arabidopsis*, NPR1 functions as a regulator of SA-mediated suppression of JA signaling and positively modulates the expression of *PR1b*, the marker gene of salicylic acid (SA) pathway. Overexpression of *OsNPR1* enhances resistance to bacterial blight, while *OsNPR1* knockdown has the opposite effect [81]. In line with that, in the current study, analysis of two marker genes involved in the SA signaling pathway, *OsPR1b* and *OsNPR1*, revealed a similar suppressive response, whereas the expression of other defense-related genes (*OsPR2*, *OsPR31*, *OsPR4*, *OsPR5*, and *OsPR10*) dramatically increased (Figure 9). Accordingly, we also observed that *OsJAZ13a* expression was increased in response to various environmental stresses, such as cold, heat shock, and salt (Figure 2).

Collectively, these observations strongly suggest that *OsJAZ13* modulates cell death in a JA/ET-dependent manner, suppresses the expression of *MYC2* and *ERF1*, gradually induces the expression of defense-related genes, and modulates the balance between defense and growth under unfavorable environmental conditions.

## 4. Materials and Methods

### 4.1. Plant Material and Growth Conditions

Rice (*Oryza sativa* L. ssp. indica) cultivars Minghui86 (MH86) were grown in a greenhouse (25 °C, 14 h light/10 h dark, 55–65% humidity) or the paddy field Experimental Station (40°06′13″N; 116°25′06″E) of the Institute of Genetics and Developmental Biology in Beijing. For the chemical treatment, seedlings at the 3-leaf stage were sprayed with 100 μM methyl-jasmonate (MeJA) or 100 μM ethephon (ETH) [42]. Solvent or sterile ddH_2_O spaying plants served as a mock control. For heat stress, the 3-leaf stage seedlings were cultured in a growth chamber, at a constant temperature of 42 °C, and with the same conditions of light and humidity as the previous greenhouse. For cold treatment, the rice seedlings were incubated at 4 ± 1 °C, in the dark, for 12 h. For the salt treatment, the roots of seedlings were soaked in 200 mM NaCl; mock-treated plants were soaked with sterile ddH_2_O [42]. Mechanical damage was done by pressing forceps into the middle of the leaf blade. Leaf tissues were collected at the indicated time, following treatment. According to the manufacturer’s instructions of TRIzol (Invitrogen, Carlsbad, USA), total RNA was isolated for gene-expression analysis.

### 4.2. Vector Construction and Plant Transformation

All primers used for vector construction are listed in Table S1. To generate gene overexpression vectors, cDNA fragments of *OsJAZ13a* (*LOC_Os10g25230*), *OsJAZ13b*, and the truncated derivative *OsJAZ13d* (amino acids 1 to 110 of OsJAZ13) were amplified from MH86 filling stage leaf, using gene-specific primers OsJAZ13aF/OsJAZ13aR, OsJAZ13bF/OsJAZ13bR and OsJAZ13dF/OsJAZ13dR, respectively. The fragments were cloned into the pMD18T-simple vector (Takara Bio, Ohtsu, Japan), digested with Bam HI/Kpn I, and ligated into pBluescript II KS (+) vector. The resulting three vectors were digested with Bam HI and then ligated into the Bam HI site of pCAMBIA1300/*Ubi* vector, generating the overexpression vectors pCAMBIA1300/*Ubi::OsJAZ13a–cis*, pCAMBIA1300/*Ubi::OsJAZ13b–cis*, and pCAMBIA1300/*Ubi::OsJAZ13d–cis*. Furthermore, *OsJAZ13a* was fused with the maize ubiquitin promoter in an antisense orientation to generate the antisense construct pCAMBIA1300*/Ubi::OsJAZ13a–trans*.

For the promoter-(β-glucuronidase) (GUS) construct, 1890 bp fragment upstream of the ATG codon of *OsJAZ13a* was amplified from BAC OSJNBA68A07 (POsJAZ13F/POsJAZ13R), ligated with pMD18T (TakaRa Bio, Ohtsu, Japan), amplified using the primers APOsJAZ13F/APOsJAZ13R, digested with Sal I/Sma I, and inserted into pCAMBIA1300-GUS vector, to generate the complete vector pCAMBIA1300/*POsJAZ13::GUS*.

The abovementioned constructs were introduced into *Agrobacterium tumefaciens* strain EHA105, and then used to transform an induced rice MH86 callus [82]. The appropriateness of all constructs was confirmed by sequencing.

### 4.3. Phylogenetic Analysis

OsJAZ13-related sequences were identified by using NCBI BLAST webpage (BLASTP and TBLASTN), with OsJAZ13 as the query sequence. Multiple alignments of 20 proteins containing the complete TIFY domain and Jas motif were performed, using Clustal W version 1.8, with default parameters. Phylogenetic trees were generated by using MEGA 4.0 software (http://www.megasoftware.net/).

### 4.4. Reverse-Transcription PCR (RT-PCR) and Real-Time Quantitative PCR (RT-qPCR)

Total RNA was extracted from approximately 100 mg of plant material, using TRIzol (Invitrogen, Carlsbad, USA) according to the manufacturer’s instructions. RNA was treated with RNase-free DNase I (Takara Bio, Ohtsu, Japan) to remove any contaminating genomic DNA. Then, cDNA was prepared from 4 μg of total RNA, using reverse-transcriptase M-MLV (Takara Bio, Ohtsu, Japan) and oligo(dT_18_) primer (Takara Bio, Ohtsu, Japan). To ensure the linear amplification, RT-PCR was performed for a limited number of cycles. *ACTIN* (*LOC_Os03g50890*) was used as an internal control for both RT-PCR and qRT-PCR. RT-qPCR analysis was performed by using the BioRad RT-PCR system and the iQTM SYBR^®^ Green Supermix (Bio-Rad, Hercules, CA, USA). Relative gene expression levels in different samples were quantified by using the 2^−ΔΔCT^ method [83]. All data are given as mean ± SE (*n* = 3) and analyzed by one-way ANOVA, followed by a Tukey’s post hoc test (SPSS 17.0, Inc., Chicago, IL, USA). The statistical significance level was set at *p* < 0.05. All RT-qPCR reactions were performed under the following conditions: initial denaturation step of 95 °C for 5 min; followed by 40 cycles of 95 °C for 20 s, 54 °C for 20 s, and 72 °C for 20 s; a melting curve analysis was performed at the end of the PCR run over the range of 65–95 °C, increasing the temperature stepwise by 0.5 °C every 10 s. The amplification efficiencies (E) of each primer pair for qRT-PCR were calculated by constructing standard curves, using a multi-point serial dilution of the cDNA template. The slope of the standard curves was used to calculate the amplification efficiency according to the following formula: E (%) = [10^(−1/slope)^ − 1] × 100. Primer pairs used for RT-PCR and RT-qPCR are listed in Table S2.

### 4.5. GUS Staining

Rice tissues from pCAMBIA1300/*POsJAZ13::GUS* transgenic plants were collected and submerged in a GUS staining solution (0.1 M sodium phosphate buffer, 10 mM ethylenediaminetetraacetic acid disodium salt, pH 7.0, 0.1% (*v/v*) Triton X-100, 5 mM potassium ferricyanide, 5 mM potassium ferrocyanide, and 1 mM 5-bromo-4-chloro-3-indolyl glucuronide). Samples were infiltrated under vacuum for 5 min and then incubated at 37 °C, in the dark, overnight. They were then de-colorized by using 70% ethanol, dissected, and photographed by using a color digital camera (DSLR-A900; SONY, Tokyo, Japan).

### 4.6. Subcellular Protein Localization Assay

For the protein localization assay, the *OsJAZ13a*, *OsJAZ13b*, *OsJAZ13c*, and *OsJAZ13d* sequences were amplified from plasmid templates, using specific primer pairs (YFPOsJAZ13aF/YFPOsJAZ13aR, YFPOsJAZ13bF/YFPOsJAZ13bR, YFPOsJAZ13cF/YFPOsJAZ13cR, and YFPOsJAZ13dF/YFPOsJAZ13dR; Table S1). The amplification products were cloned into pAVA321 digested with Xho I, to generate pAVA321-*OsJAZ13a::YFP* to pAVA321-*OsJAZ13d::YFP*, in which the *OsJAZ13a–d* coding sequences lacking the end codon were fused in-frame with the sequence for the yellow fluorescent protein (YFP), accordingly. These constructs were then used to transform rice protoplasts, using the polyethylene glycol method [84]. After 20–24 h incubation at 28 °C in the dark, YFP fluorescence was visualized by a confocal laser scanning microscope (Leica TCS SP5, Leica Microsystems, Mannheim, Germany).

### 4.7. Y2H Assay

The coding sequences of *OsJAZ13a*, *OsJAZ13b*, *OsJAZ13c*, *OsJAZ13d*, *OsCOI1* (*LOC_Os01g63420*), N-terminus of *OsMYC2* (*LOC_Os10g42430*), and C-termini of *OsMYC2*, *OsNINJA* (*LOC_Os05g48500*), and *OsTPL* (*LOC_Os03g14980*) were amplified from MH86 cDNA and cloned into pGBKT7 and pGADT7 vectors (Clontech, Palo Alto, CA, USA), accordingly. Primers used for the preparation of Y2H constructs are listed in Table S1. The bait and prey constructs were then used to transform yeast strain Y2Hgold (Clontech, Palo Alto, CA, USA), following the manufacturer’s instructions. All transformants were grown on synthetic defined (SD) medium lacking Ade, His, Leu, and Trp. For assaying the OsCOI1–OsJAZ13 interaction, the co-transformed cells were grown on the SD/-Ade/-His/-Leu/-Trp dropout medium, with or without 30 μM COR.

### 4.8. Growth Inhibition Assay

For the root growth assay, T_1_ or T_2_ seeds of positive transformants were sterilized and placed on half-strength Murashige and Skoog medium (1/2MS) plates containing 30 mg/L hygromycin and grown (14 h light/10 h dark, 28 °C) for 2 days. The negative control (WT) seeds were placed on the same plates, but without hygromycin. After 2 days, hygromycin-resistant seedlings and control plants were transferred onto fresh 1/2MS medium containing 20 μM MeJA or without MeJA, for a further 7 days. The root and shoot lengths were then determined and photographed, using a color digital camera (DSLR-A900; SONY, Tokyo, Japan). These experiments were performed for three independent biological repeats. Error bars represent SD. Student’s *t*-test was used for statistical analysis.

### 4.9. In Vivo Degradation of JAZ-YFP Fusion Proteins

Transgenic seeds producing OsJAZ13a-YFP, OsJAZ13b-YFP, and OsJAZ13d-YFP were grown on 1/2MS plates supplemented with 30 mg/L hygromycin, at 28 °C, for 2 days, when the root length reached up to 0.5 cm. They were then transferred to liquid 1/2 MS medium, with or without 50 μM MeJA, and incubated for 2 h, at room temperature. The root tips were cut and placed on a glass slide, and YFP fluorescence was analyzed under a confocal laser scanning microscope (Leica TCS SP5, Leica Microsystems, Mannheim, Germany), using imaging software provided by the manufacturer. In a parallel experiment, seedlings were pretreated with water or 100 μM MG132 (Sigma-Aldrich, St. Louis, Missouri, USA) for 2 h prior to the addition of 50 μM MeJA. Images were shown after the same exposure time and were acquired by using the same microscope parameters.

### 4.10. Staining with 3,3′-Diaminobenzidine (DAB)

For the DAB staining experiment, H_2_O_2_ levels in the leaf were determined by DAB staining, as described previously [5], with some modifications. Briefly, transgenic plants were grown in a controlled culture room, at 25 °C, with a relative humidity of 55–65%, and under long-day conditions (14 h light/10 h dark), with white light illumination. Then, 30–45 day leaves were detached until brown lesion mimics emerged in the sheath. An approximately 5 cm leaf blade (5 cm away from the leaf tip) was detached and vacuum-infiltrated with the DAB staining solution (0.5 mg/mL, pH 3.8) for 30 min, and then it was exposed to continuous white light for 8 h, at 28 °C. The leaves were then de-colorized by soaking in 95 °C ethanol for 15 min. The procedure was repeated three to four times; H_2_O_2_ accumulation was determined as brown DAB precipitate on the leaf.

### 4.11. Trypan Blue Staining

Dead cells in the leaf were visualized by trypan blue staining, as described previously [85], with few modifications. The same leaf was analyzed as that used for DAB staining. Briefly, an approximately 5 cm leaf blade (10 cm away from the leaf tip) was detached and submerged in a lactophenol–trypan blue solution (2.5 mg/mL trypan blue, 25% (*w/v*) lactic acid, 23% water-saturated phenol, and 25% glycerol) preheated to 70 °C, infiltrated for 10 min, then heated over boiling water for 2 min, and stained overnight, at room temperature. After de-staining in chloral hydrate solution (25 g in 10 mL of H_2_O), to reduce background staining, leaf images were acquired by using a color digital camera (DSLR-A900; SONY, Tokyo, Japan).

## Supplementary Materials

Supplementary materials can be found at https://www.mdpi.com/1422-0067/21/12/4379/s1.

## Abbreviations

1/2MS	Half-strength Murashige and Skoog medium (1/2MS)
AS	Alternative splicing
bHLH	Basic helix-loop-helix
COI1	CORONATINE INSENSITIVE1
DAB	3,3′-diaminobenzidine
EAR	ERF-associated amphiphilic repression
ET	Ethylene
ETH	Ethephon
GUS	Glucuronidase
JA	Jasmonate
JA-Ile	Jasmonoyl-l-isoleucine
JAZ	Jasmonate zim-domain
MeJA	Methyl jasmonate
NINJA	NOVEL INTERACTOR OF JAZ
ORF	Open reading frame
PR	Pathogenesis-related
ROS	Reactive oxygen species
RT-PCR	Reverse transcription-PCR
RT-qPCR	Real-time quantitative PCR
SA	Salicylic acid
SCF	SKP1-CUL1-F-box protein
SD	Synthetic defined
TF	Transcription factor
TPL	TOPLESS
WT	Wild type
Y2H	Yeast two-hybrid
YFP	Yellow fluorescent protein
