# Supplementary material for: OsJAZ13 Negatively Regulates Jasmonate Signaling and Activates Hypersensitive Cell Death Response in Rice
Source: Int J Mol Sci. 2020 Jun 19;21(12):4379. doi: 10.3390/ijms21124379 (PMC7352843; doi:10.3390/ijms21124379)
Supplement: Supplementary file 1 [file ijms-21-04379-s001.pdf]

Figure S1

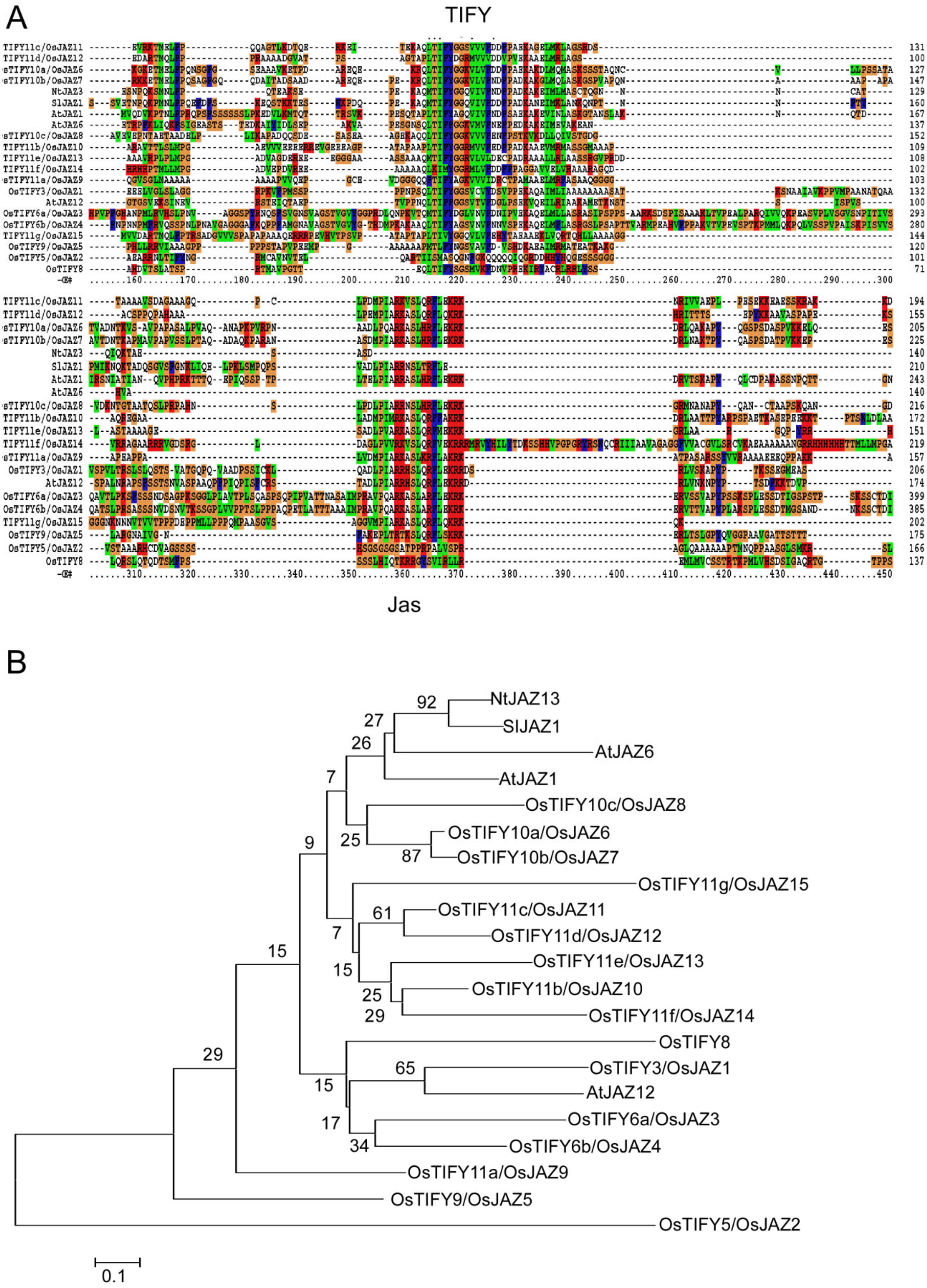

Figure S1. Amino acid sequence and phylogenetic analysis of rice, *Arabidopsis*, tomato, and tobacco JAZ proteins. (A) Amino acid sequences were aligned with ClustalW 1.8. TIFY and Jas indicate the conserved domain of JAZ protein. The incolor alignment depicts amino acids with similar hydrophobicity and was generated. (B) The phylogenetic tree was constructed with MEGA 4.0 using

the neighbor-joining method. The accession number of these proteins are as follows: OsTIFY3/OsJAZ1 (CAE03550.1), OsTIFY5/OsJAZ2 (NP_001058910.2), OsTIFY6a/OsJAZ3 (NP_001061849.1), OsTIFY6b/OsJAZ4 (NP_001063121.1), OsTIFY8 (NP_001048026.1), OsTIFY9/OsJAZ5 (NP_001052661.1), OsTIFY10a/OsJAZ6 (NP_001050322.1), OsTIFY10b/OsJAZ7 (NP_001060268.1), OsTIFY10c/OsJAZ8 (NP_001063273.1), OsTIFY11a/OsJAZ9 (NP_001049166.1), OsTIFY11b (NP_001049168.1), OsTIFY11c/OsJAZ11 (NP_001049167.1), OsTIFY11d/OsJAZ12 (NP_001064513.1), OsTIFY11e/OsJAZ13 (NP_001064511.1), OsTIFY11f/OsJAZ14 (AAP53565.1), and OsTIFY11g/OsJAZ15 (NP_001173460.1) are from rice; AtJAZ1 (NP_564075.1), AtJAZ6 (NP_565043.1), and AtJAZ12 (NP_197590.1) are from Arabidopsis; NtJAZ3 (BAG68657.1) is from tobacco; SlJAZ1 (ABU88421.1) is from tomato;

Supplementary Table S1 Primers used for gene clone and plasmid construction

Gene	Primer pairs 5’-3’		Tm (°C)		Fragment size (bp)
	Forward primer	Reverse primer	Forward primer	Reverse primer	
**Gene clone**					
OsJAZ13a	AAGAAGAAATTAACCCGAG	GGATTAAAGCTGACGTGT	51.2	50.8	628
OsJAZ13b	AAGAAGAAATTAACCCGAG	GGATTAAAGCTGACGTGT	51.2	50.8	474
OsJAZ13c	AAGAAGAAATTAACCCGAG	TTATGGGATTAAAGCTGACG	51.2	55.5	380
OsNINJA	ACATGGACGATGAGAATG	CATTGAAACCGTCATACAG	52.1	52.2	1386
OsCOI1	CCTGGTTCGGGCTAGATC	ACAATCACGCAGGATGCA	58.2	59.8	1855
OsMYC2	ATGAACCTTTGGACGGACGA	TTGGGTTTCCTCGCTAGA	62.8	56.9	2142
OsTPL	TTCTGTGACCTGTAGAATAGGGTAG	TGGTTAGTGAATTTGTACCGTG	58	57.6	3553
**Overexpression and antisense**					
Ox-OsJAZ13a	CGGGATCCAAGAAGAAATTAACCCGAG	GGGGTACCGGATTAAAGCTGACGTGT	51.2	50.8	644
Ox-OsJAZ13b	CGGGATCCAAGAAGAAATTAACCCGAG	GGGGTACCGGATTAAAGCTGACGTGT	51.2	50.8	490
Ox-AOsJAZ13c	CGGGATCCAAGAAGAAATTAACCCGAG	GGGGTACCTTATGGGATTAAAGCTGACG	51.2	55.5	396
Ox-AOsJAZ13d	CGGGATCCAAGAAGAAATTAACCCGAG	GGGGTACCTTACAGGTCCGCCGACTC	51.2	59.8	346
Anti-OsJAZ13	CGGGATCCAAGAAGAAATTAACCCGAG	GGGGTACCGGATTAAAGCTGACGTGT	51.2	50.8	644
**Overexpression fusion OsJAZ13 with YFP**					
YFP-Ox-OsJAZ13a	CGGGATCCATGGCGGCGGAGGCGGC	GAGCTCTCTAGATAGATCTGAGTCCGGAC	74.4	56.7	1244
YFP-Ox-OsJAZ13b	CGGGATCCATGGCGGCGGAGGCGGC	GAGCTCTCTAGATAGATCTGAGTCCGGAC	74.4	56.7	1094
YFP-Ox-OsJAZ13d	CGGGATCCATGGCGGCGGAGGCGGC	GAGCTCTCTACAGGTCCGCCGACTC	74.4	61.4	1076
**Subcellular location**					
YFPOsJAZ13a	CATGCCATGGCGGCGGAGGCGGC	CATGCCATGGAGAGCGCGAGCGCGA	74.4	72.2	511
YFPOsJAZ13b	CATGCCATGGCGGCGGAGGCGGC	CATGCCATGGAGAGCGCGAGCGCGA	74.4	72.2	365
YFPOsJAZ13c	CATGCCATGGCGGCGGAGGCGGC	CATGCCATGGATGGGATTAAAGCTGACGTG	74.4	65.3	293
YFPOsJAZ13d	CATGCCATGGCGGCGGAGGCGGC	CATGCCATGGAGAGCGCGAGCGCGA	74.4	72.2	347
**Y2H assay**					
Y2HOsJAZ13a	GAATTCATGGCGGCGGAGGCG	CGGGATCCCGAGCGCGAGCGCGAG	68.2	69.5	513
Y2HOsJAZ13b	GAATTCATGGCGGCGGAGGCG	CGGGATCCCGAGCGCGAGCGCGAG	68.2	69.5	363
Y2HOsJAZ13c	GAATTCATGGCGGCGGAGGCG	CGGGATCCCTGGGATTAAAGCTGACG	68.2	54.3	291
Y2HOsJAZ13d	GAATTCATGGCGGCGGAGGCG	CGGGATCCCGGCGAGATCGTCGCG	68.2	66.5	345
Y2HBDCOI	GAATTCATGGGTGGCGAGGTGC	GGATCCCTCACGCAGGATGCAAG	61.8	57.4	1801
Y2HBDOsMYC2	CGGAATTCATGAACCTTTGGACGGA	CCCCGGGTTACCGGGCGGCGGTG	54.6	67.6	2157
Y2HNADMYC2	CGGAATTCATGAACCTTTGGACGGA	CCCCGGGTTACTGCTGCTGCTGCTG	54.6	57.5	1044
Y2HCADMYC2	CGGAATTCATGAAGGACTCCATCTCCG	CCCCGGGTTACCGGGCGGCGGTG	57.6	67.6	1278
Y2HOsTPL	CGGAATTCATGTCGTCGCTGAGCCGG	GAAGATCTTTATCTTTCTGGTTGATCAGAACTTG	66	60.1	3418
Y2HADNINJA	CGGAATTCATGGACGATGAGAATGGC	GAAGATCTTTAGTTTTGGGCTGAGGC	56.8	56.4	1372
**Gus promoter**					
POsJAZ13	TCTGAAGCCGACGCCT	ACCAGCCGCTGCTTG	59.1	57.9	1890
APOsJAZ13	ACGCGTCGACTCTGAAGCCGACGCCT	TCCCCCGGGACCAGCCGCTGCTTG	59.1	57.9	1909

Note: underlined sequences indicate restriction enzyme sites.

Supplementary Table S2 Primers used for RT-PCR and qRT-PCR

Gene	Primer pairs 5'-3'		Tm (°C)		Fragment size (bp)	E (%)
	Forward primer	Reverse primer	Forward primer	Reverse primer		
**RT-PCR**						
OsJAZ13	AAGAAGAAATTAACCCGAG	TTATGGGATTAAAGCTGACG	51.2	55.5	632	-
HSR203J	CGTACGTACGGCAGCAATG	TGTACGGGTGGTCCTTGG	61.3	60.4	833	-
ACTIN	TCATGAAGATCCTGACGGAG	AACAGCTCCTCTTGGCTTAG	58.7	56.5	602	-
**qRT-PCR**						
qOsMYC2	AGGACCTCATGATCCAGCA	TTGGGTTTCCTCGCTAGA	59.1	56.9	140	94.2
qOsEIL1	GAGGTGGTCTGGTGATGGA	CGAAGCCGAGGTTCTTGTC	59.4	60.9	99	91.6
qOsERF1	ACGCACAGTGAAGCAAGCA	GCCTGATCCCTTTGCCGTC	61.8	65.3	129	90.6
qOsERF047	TCTTCCTGCTGCGATGTATG	AACACGGGTAGGAGAAGGAC	60	58.1	92	103.1
qOsERF077	ATCTGAGCCTCGGGATGT	GAGCTACCTCCTTCTTGACG	61.5	56.7	138	101.8
qOsERF130	ACGTACGTGAGTAGAGCATCA	TGCTCTCCACTCCCACAT	56	57.5	102	93.1
qOsNPR1	AAACCGGATCAGTTTCATCA	AAGAACACTTAGCTCGGATGAC	58	57.6	117	101.3
qOsPR1b	GGCAACTTCGTCGGACAGA	CCGTGGACCTGTTTACATTTTCA	62.4	63.1	122	103.7
qOsPR2	CGATTACGTTTCCTCCGA	GGGCCATACAGAGTACATGC	57.2	58	121	95.7
qOsPR31	GAGCTATAGCCTCAACCTCA	TGCGTATGAACGGATCTC	54.5	55.5	120	96.1
qOsPR4	AGCGCATATTGTGCCACATG	GGATACACTTGCCACACGAGTCT	63	62.7	121	96.3
qOsPR5	CAACAGCAACTACCAAGTCGTCTT	CAAGGTGTCGTTTTATTCATCAACTTT	61.9	62.7	125	92.7
qOsPR5-Like	GCAACAGAAACACTAACACG	TGTACCCGCACTTGTTGA	54.4	57	135	95.7
qOsPR10	CCCTGCCGAATACGCCTAA	CTCAAACGCCACgAgAATTTG	63.2	62.9	121	102.2
qOsAOS1	CCTGTTCGGCCTCATGGAGAA	GGAGCGAGTGGAGGAGCGT	66.9	65.3	239	104.6
qOsAOC1	CAGATCGAGCTGCCTGAT	GCAGGGAGGAAACAAATATC	57.5	56.2	108	97.0
QNR2	TGGAGAAGATGGGCTATGA	ACTCGAGAGAGCTCCAAACT	56.7	55.9	142	95.9
qNADP-ME	AGCCTATGACCTTGGATTG	AGCCAAATGAAGCTGTACAC	55.1	55.5	139	97.6

Note: TM: melting temperatures; E: PCR efficiency (%)
